# Evaluating Pilot Implementation of ‘PenCS Flu Topbar’ App in Medical Practices to Improve National Immunisation Program–Funded Seasonal Influenza Vaccination in Central Queensland, Australia

**DOI:** 10.1111/irv.13280

**Published:** 2024-04-16

**Authors:** Gulam Khandaker, Gwenda Chapman, Arifuzzaman Khan, Mahmudul Hassan Al Imam, Robert Menzies, Nicolas Smoll, Jacina Walker, Michael Kirk, Kerrie Wiley

**Affiliations:** ^1^ Central Queensland Public Health Unit Central Queensland Hospital and Health Service Rockhampton Queensland Australia; ^2^ Research Division Central Queensland University Rockhampton Queensland Australia; ^3^ Discipline of Child and Adolescent Health, Sydney Medical School The University of Sydney Camperdown New South Wales Australia; ^4^ Herston Biofabrication Institute Metro North Health Herston Queensland Australia; ^5^ Wide Bay Public Health Unit Hervey Bay Hospital and Health Service Hervey Bay Queensland Australia; ^6^ School of Public Health The University of Queensland Herston Queensland Australia; ^7^ School of Health, Medical and Applied Sciences Central Queensland University Rockhampton Queensland Australia; ^8^ Research Division Sanofi Pasteur Canterbury New South Wales Australia; ^9^ Sunshine Coast Public Health Unit Sunshine Coast Hospital and Health Service Maroochydore Queensland Australia; ^10^ Rockhampton Business Unit Central Queensland Hospital and Health Service Rockhampton Queensland Australia; ^11^ Sydney School of Public Health The University of Sydney Camperdown New South Wales Australia; ^12^ Sydney Infectious Diseases Institute The University of Sydney Camperdown New South Wales Australia

**Keywords:** application, clinical decision support system, health information technology, influenza, vaccination

## Abstract

**Background:**

The ‘PenCS Flu Topbar’ app was deployed in Central Queensland (CQ), Australia, medical practices through a pilot programme in March 2021.

**Methods:**

We evaluated the app's user experience and examined whether the introduction of ‘PenCS Flu Topbar’ in medical practices could improve the coverage of NIP‐funded influenza vaccinations. We conducted a mixed‐method study including a qualitative analysis of in‐depth interviews with key end‐users and a quantitative analysis of influenza vaccine administrative data.

**Results:**

‘PenCS Flu Topbar’ app users reported positive experiences identifying patients eligible for NIP‐funded seasonal influenza vaccination. A total of 3606 NIP‐funded influenza vaccinations was administered in the eight intervention practices, 14% higher than the eight control practices. NIP‐funded vaccination coverage within practices was significantly higher in the intervention practices (31.2%) than in the control practices (27.3%) (absolute difference: 3.9%; 95% CI: 2.9%–5.0%; *p* < 0.001). The coverage was substantially higher in Aboriginal and Torres Strait Islander people aged more than 6 months, pregnant women and children aged 6 months to less than 5 years for the practices where the app was introduced when compared to control practices: incidence rate ratio (IRR) 2.4 (95% CI: 1.8–3.2), IRR 2.7 (95% CI: 1.8–4.2) and IRR 2.3 (1.8–2.9) times higher, respectively.

**Conclusions:**

Our evaluation indicated that the ‘PenCS Flu Topbar’ app is useful for identifying the patients eligible for NIP‐funded influenza vaccination and is likely to increase NIP‐funded influenza vaccine coverage in the eligible populations. Future impact evaluation including a greater number of practices and a wider geographical area is essential.

## 1 Introduction

Influenza remains one of the leading vaccine preventable diseases worldwide, often causing serious illness and death in vulnerable populations, with the elderly and people with underlying medical conditions or chronic diseases at greater risk. The most recent global disease burden study in 2017 estimates that influenza is associated with 9.4 million hospitalisations resulting in 145,000 (95% uncertainty interval: 99,000–200,000) deaths each year [1]. In Australia, influenza accounts for one‐third of the total vaccine preventable diseases contributing to 5674 disability‐adjusted life years [2]. The Australian Department of Health (DOH) considers vaccination as the most effective tool for reducing the burden of influenza. Improved monitoring and uptake of influenza vaccine is a priority of Australia's national immunisation strategy [3].

Annual influenza vaccination is recommended under the National Immunisation Program (NIP) for vulnerable groups including people aged 65 years and above, pregnant women, Aboriginal and Torres Strait Islander people aged 6 months and over, children aged 6 months to 5 years and people with chronic medical conditions [4]. However, vaccination coverage among these groups is suboptimal. While the overall immunisation coverage among children aged less than 5 years is more than 95% in Australia, the influenza vaccination coverage is only 39.7% for children aged between 6 months and less than 5 years [5]. Moreover, the seasonal influenza vaccine coverage of adults over 65 years is only 58.0% [5]. Although the coverage has been increasing gradually since 2020 [6, 7], clinicians and public health programmes need to focus on enhancing strategies to improve vaccination rates to decrease the disease burden.

Utilising health information technology (IT) is an effective strategy to improve vaccine communication and coverage [8]. Health IT interventions can facilitate the rapid or real‐time identification of individuals in need of vaccination and provide the foundation for vaccine‐oriented parental communication or clinical alerts in a flexible and tailored manner [8, 9]. General practitioners (GPs) are familiar with using health IT assisting clinical decision support systems (CDSSs), such as ‘PenCS Topbar’ [10] to screen for chronic diseases and make evidence‐based clinical decisions. CDSS systems have been shown to increase influenza vaccination coverage in both adults and children in America [11]. Therefore, it is imperative to evaluate the outcome of CDSS technology in improving vaccination coverage in regional Australia.

The PenCS Flu Topbar app is specifically designed to help identify patients who are recommended for influenza vaccination [10, 12]. The app assists medical practices in identifying patients eligible for NIP‐funded influenza vaccinations. The app was deployed in Central Queensland (CQ), Australia, medical practices through a pilot programme in March 2021. In this context, we aimed to evaluate the user experience and immediate impact of this pilot implementation of ‘PenCS Flu Topbar’ in increasing the NIP‐funded influenza coverage.

## 2 Methods

A mixed‐method design including precomparison and postcomparison analysis of qualitative and quantitative data was conducted to evaluate the process, user insights and immediate impact of the pilot implementation of ‘PenCS Flu Topbar’ to improve the coverage of NIP‐funded influenza vaccinations in selected medical practices in CQ. For the qualitative study, the pre‐implementation phase refers to the period before the intervention (was first introduced on 29/2/2023) was implemented, while the postimplementation phase refers to the period after the intervention was introduced in the study area. The pre‐implementation interviews were conducted between 15/3/2021 and 19/3/2021, and the postimplementation interviews were conducted between 23/08/2021 and 31/08/2021. For the quantitative study, the vaccination data were collected for the years 2018 to 2021, from March to August, from eight intervention practices and eight control practices. Therefore, the pre‐implementation phase for the quantitative study is the period from 2018 to 2020, while the postimplementation phase is the year 2021.

**FIGURE 1:**
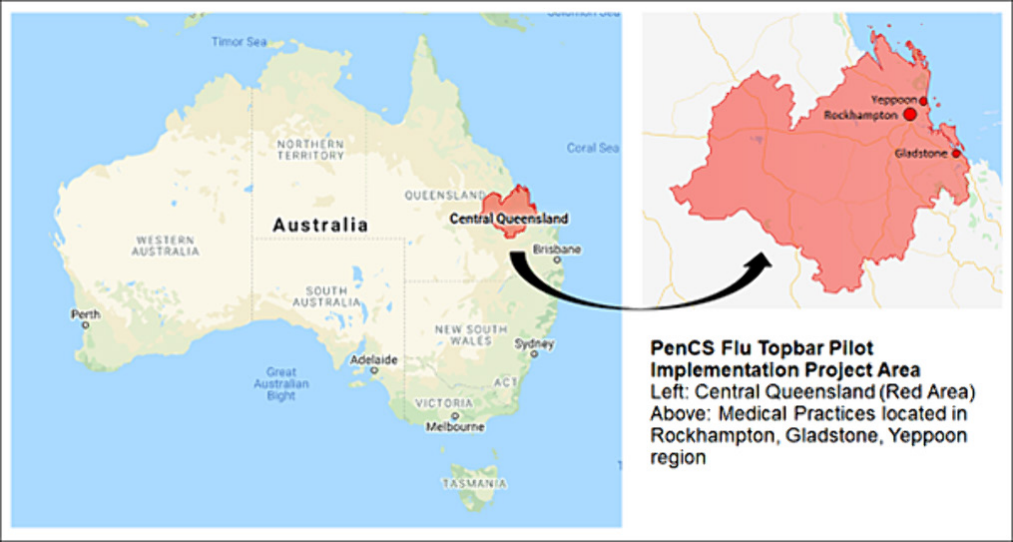
‘PenCS Flu Topbar’ implementation area.

The study was conducted in the CQ region (Figure 1) which covers 117,588 km^2^ and a population of 234,179. Approximately 14% of the population is aged over 65 years, and 6% of the population is Aboriginal and/or Torres Strait Islanders [13]. There are 63 medical practices in this region providing primary health care. Key participants from the medical practices included GPs, practice managers (PMs) and practice nurses (PNs).

The intervention in this study was the ‘PenCS Flu Topbar’ CDSS app. The app was implemented in 36 medical practices in CQ as a pilot programme prior to the peak of the 2021 influenza season (March to April in 2021). When the healthcare provider opens a patient record in their clinical system, the Topbar will check the patient's details (age, ethnicity, measurements and clinical history) against the NIP criteria for influenza vaccine eligibility. Depending on the patient, a notification will appear in Topbar suggesting that the patient has not received an influenza vaccine in the current season, displaying different coloured notifications depending on the patient's NIP eligibility.

Prior to the implementation of ‘PenCS Flu Topbar’ (March 2021), a convenience sample of eight medical practices and Aboriginal community controlled health services (ACCHS) was sought for insight into their current practices. Participants from these practices along with participants from a further three purposively sought practices who used the ‘PenCS Flu Topbar’ app were interviewed after implementation (August 2021), giving a total of 11 practices included in the study. Qualitative in‐depth interviews with the practice GPs, PMs and PNs were conducted following a semi‐structured interview guide exploring the process practices used to identify NIP‐eligible patients and the involvement of software in this process, before and after the intervention (see Data S1). The interviews were recorded with permission from the participants. The audio files were transcribed by a confidential transcription service, and the de‐identified transcripts were used in the analysis. Data were thematically analysed using a framework approach [14]. Two independent reviewers under the guidance of a senior reviewer coded and recoded the interview data based on the framework that was developed deductively and inductively. The themes emerging from the framework were summarised and reported as a narrative interpretation.

As part of the quantitative evaluation, a convenience sample of eight intervention practices who agreed to participate in the study and where ‘PenCS Flu Topbar’ was implemented, and eight control practices without the implementation were compared. De‐identified aggregated data detailing the number of visits and influenza vaccinations during the influenza seasons (March to August) of the year 2018 to 2021 were collected from the eight intervention practices and eight control practices (see Data S2 and S3). The data were imported into analysis software R version 4.1.1 [15] for cleaning, manipulation and analysis. Comparative summary statistics were produced comparing the numbers of visits and vaccinations by eligible and non‐eligible NIP‐funded influenza vaccination in intervention and control practices for 2021. A *‘*Two‐Proportions Z‐Test’ was used to evaluate the statistical differences between the proportion of the vaccinations in intervention and control practices. The incidence rate ratio, along with its 95% confidence intervals (CI), was estimated from the vaccination coverage (%) among the number of visits to the practices. The relative differences in vaccination coverage were then compared between intervention and control practices. Subgroup analyses were performed using above mentioned statistical approaches in different populations eligible for NIP‐funded influenza vaccination including the following age groups: people aged 65 years and older, children of 6 months to less than 5 years, Aboriginal and Torres Strait Islanders more than six months of age and pregnant women.

Written informed consent was obtained from all participants. Ethical approval was obtained from the Townsville Hospital and Health Service Human Research Ethics Committee (HREC) (HREC/QTHS/73995 and HREC/QTHS/74743).

## 3 Results

### 3.1 Characteristics of the Participating Medical Practices

Table 1 provides a summary of the characteristics of the eleven medical practices that took part in this study, comparing them to non‐participating practices in CQ. On average, each medical practice employed four doctors. All 11 participating practices offered vaccination services, two were exclusively dedicated to Aboriginal Medical Services and eight were situated in Inner Regional Australia. Notably, the characteristics of the participating medical practices did not show significant differences from those of the nonparticipating practices.

**TABLE 1:** Characteristics of medical practices in CQ.

Characteristics	Participating medical practice *N* = 11		Non‐participating medical practice *N* = 52		*p* Value
	*n*	%	*n*	%	
Number of doctors per medical practice (mean ± SD)	4 ± 3		4 ± 3		0.852^a^
Aboriginal medical service					
Yes	2	18.2	50	96.2	0.146^b^
No	9	81.8	2	3.8	
Vaccine service provider					
Yes	11	100.0	48	92.3	0.588^b^
No	0	0.0	4	7.7	
Remoteness index of Australia					
Inner regional	8	72.7	35	67.3	0.892^b^
Outer regional	2	18.2	13	25.0	
Remote	1	9.1	4	7.7	

^a^ *t*‐test.

^b^ Chi‐squared test with Monte Carlo simulation.

### 3.2 Qualitative Exploration

The 11 practices from the CQ area included practices in Rockhampton, Yeppoon, Gladstone and Mount Morgan (Table 2). Use of the different Topbars, which are CDSSs designed to assist medical practitioners at the point of care (to which the ‘Flu Topbar app’ was an add‐on), varied across practices, ranging from daily use to little or no use at all. Based on the patterns of use described, a typology of user profiles was developed, whereby practices could be categorised as either an *Active User practice*, an *Occasional User practice* or an *Inactive or Non‐User practice* (Table 3). Of note, the profiles of the practices that were included in both the pre‐implementation and postimplementation phases of the study did not change postimplementation of the app.

**TABLE 2:** Participating practice characteristics and data collection.

Practice	Practice employees interviewed	Practice Topbar user type
1	1 practice manager^a^	Non‐User practice
	1 GP^a^	
2	1 practice manager^a^	Occasional User practice
3	1 practice manager^a^	Occasional User practice
4	1 practice manager^a^	Active User practice
	1 practice nurse^b^	
	GP 1^b^	
	GP 2^c^	
5	1 practice manager^b^	Occasional User practice
	1 practice nurse^a^	
6	1 practice manager^a^	Non‐User practice
	1 GP^a^	
7	1 GP^a^	Occasional User practice
8	1 practice manager^a^	Non‐User practice
	1 practice nurse^b^	
9	1 practice manager^c^	Active User practice
	1 practice nurse^c^	
10	1 practice manager^c^	Active User practice
11	1 practice manager^c^	Occasional User practice

^a^ Participant took part in both pre‐ and post‐app implementation interview.

^b^ Participant took part in pre‐app implementation interview only.

^c^ Participant took part in post‐app implementation interview only.

**TABLE 3:** ‘PenCS Flu Topbar’ user profiles.

Typology	Characteristic
Active user	Practice champion—usually practice manager or practice nurse Engages with Topbar daily/several times per week Incorporates use of Topbar with each patient Training and advocates to all staff in practice to use Topbar with every patient (receptionists, nurses and GPs) Uses include competing missing data (e.g., weight and waist circumference), reporting health outcomes and prompting clinical reminders
Occasional user	Weekly to monthly use Have a ‘blitz’ to complete missing data, or to meet key performance indicators (KPI) Driven by reporting requirements
Inactive or non‐user	Low to no use Some report previous technical glitches Time constraints commonly reported as a barrier to using Some users have not heard of software/unable to locate on desktop Rely on other practice software to collect data for mandatory reporting (e.g., best practice)

#### 3.2.1 Findings From the Pre‐App Implementation Process Evaluation

Prior to the implementation of the ‘PenCS Flu Topbar’ app, most of the practices had similar processes. Patients were identified as eligible for NIP‐funded influenza vaccination through a combination of the following:

- The patient actively requesting a vaccine appointment, often after seeing clinic advertisements that the vaccine is available;
- Reception staff identifying the patient as eligible through age/care plan visible in the patient record when they present, and flagging it in the record for the doctor;
- The doctor having knowledge of the patient's history of flu vaccination/existing chronic conditions/eligibility criteria.

The two Aboriginal community controlled services had slightly different processes, as most of their patient populations were eligible for NIP‐funded influenza vaccines. These practices run annual influenza immunisation clinics. Some clinics had recall/reminder systems in place using their main practice software, but these relied on prior vaccines being entered manually into the system to enable the reminder for the next visit.

Medical practices (those who were interviewed) used different apps like Topbars [16] for various purposes, ranging from daily use for key performance indicators and data quality to intermittent use for reporting and data mining purposes, and some practices reported nonuse:

I use it every day; every single day I use PenCS for one reason or another …. I have learned PenCS back to front and like I said, since they've upgraded it, it's a lot easier to use. (PM, active user)

Sometimes we realise ooh, we haven't got enough information for this, and PenCS gives you a quick overall look …. and then we have a blitz on making sure we've got everybody's allergies in, for example, that sort of thing. (PM, occasional user)

Some GPs and PNs reported using different Topbar apps, and many reported not using it, with some unaware of how to access it.

I never [use Topbar], that's why I was looking honestly, to find out where [it is located]. (GP, non‐user)

Some medical practices reported technical difficulties with the apps intermittently ‘dropping out’ or being ‘glitchy’, and some of those reporting these issues associated them temporally with recent software updates. One practice reported that Topbar ‘completely disappeared’, while another reported the software ‘crashing’ their entire system, leading them to minimise using it, as it was costing them money in the required IT support to get their system running again. The majority of practices used different apps than PenCS Topbars [16] called ‘Best Practice’, some used ‘Medical Director’ and one used ‘Communicare’ for documentation and management of patient data.

#### 3.2.2 User Experiences of the ‘PenCS Flu Topbar’: Findings From the Post‐App Implementation Interviews

All practices reported a range of challenges during the 2021 influenza immunisation programme related to the COVID‐19 vaccine rollout. Active users' practices reported using ‘PenCS Flu Topbar’ regularly whereas inactive and non‐Topbar users did not report using the Flu app. Active users reported benefits of using ‘PenCS Flu Topbar’ including improved efficacy for identifying eligible patients with chronic diseases and ensuring consistency of care of patients. One PM gave an example of identifying patients eligible for the NIP‐funded vaccine demonstrating a case of 50‐year‐old with high blood pressure:

The GPs know that over 65 are eligible, but not other conditions, the chronic conditions. This is where the prompts are handy. (PM, active user)

Another explained how it empowered patients and improved care stating:

Patients don't know if they are eligible, if the prompts say they are, then they know, and it becomes part of their annual care plan. (PM, active Topbar user)

Overall users found the software easy to navigate and complemented existing processes. ‘PenCS Flu Topbar’ app users did not report any challenges with implementation.

Easy, really good. When you click and bring it up on the screen, you can see everything. Everything is there in tick boxes. (PN, active Topbar user)

### 3.3 Impact Evaluation

#### 3.3.1 Impact on Overall NIP‐Funded Vaccination Coverage

NIP‐funded vaccination coverage within practices was significantly higher in the intervention practices than in the control practices (31.2% vs. 27.3%, respectively; *p* < 0.001). Conversely, we found a significantly lower coverage of non–NIP‐funded vaccinations in the intervention practices than in the coverage observed in the control practices (13.5% vs. 21.7%, respectively; *p* < 0.001). Also, we estimated the incidence rate ratio of the vaccination uptake between intervention and control practices. We found that NIP‐funded vaccination coverage is 14% higher in the intervention practices when compared to the control practices. On the contrary, we found lower coverage of non–NIP‐funded vaccination in the intervention practices when compared to control practices by 38.2% (Table 4).

**TABLE 4:** Overall vaccination coverage and relative difference of the coverage between intervention and control.

Measures	Intervention	Control	*p* Value
Number of NIP eligible visits in the practice	11,544	15,618	
Numbers of NIP‐funded vaccinations in the practice in the practice	3606	4264	
NIP‐funded vaccine coverage in % (95%CI)	31.2% (30.4%–32.1%)	27.3% (26.6%–28.0%)	
Incidence rate ratio (intervention/control) of vaccination coverage (95% CI) for NIP‐funded vaccination	1.144 (1.094–1.196)		< 0.001
Relative change in % (95% CI) of NIP‐funded vaccination in intervention in comparison to control	14% (9.4%–19.6%)		
Number of non‐NIP eligible visits in the practice	1075	1368	
Numbers of non–NIP‐funded vaccinations in the practice	7978	6309	
Non–NIP‐funded vaccine coverage in % (95% CI)	13.5% (12.7%–14.2%)	21.7% (20.7%–22.7%)	
Incidence rate ratio (intervention/control) of vaccination coverage (95% CI) for non–NIP‐funded vaccination	0.62 (0.573–0.672)		< 0.001
Relative change in % (95% CI) of non–NIP‐funded vaccination in % (95% CI) in intervention in comparison to control	–38.2% (–42.7% to –32.8%)		

Abbreviations: NIP, National Immunisation Program; 95% CI, 95% confidence interval.

#### 3.3.2 Impact on Overall NIP‐Funded Vaccination Coverage in Different Subgroups Eligible for NIP‐Funded Influenza Vaccinations

The highest number of vaccinations was administered for 65 years and older age group in both intervention and control practices. The NIP‐funded vaccination coverage was significantly higher in the intervention practices than in the control practices (55.0% vs. 50.2%, respectively; *p* = 0.006) in 65 years and older age group. In 6 months to less than 5 years age group, the NIP‐funded vaccination coverage was significantly higher in the intervention practices than in the control practices (19.9% vs. 8.5%, respectively; *p* < 0.001). Among Aboriginal and Torres Strait Islanders of more than 6 months of age, the NIP‐funded vaccination coverage was significantly higher in the intervention practices than in the control practices (35.3% vs. 14.5%, respectively; *p* < 0.001) (Table 5).

**TABLE 5:** Vaccination coverage and relative difference of the coverages in eligible subgroup population between intervention and control.

Measures	Intervention practice (*N* = 8)	Control practice (*N* = 8)	*p* Value
A. Eligible group: 65 years and older			
Number of visits to the practice	4822	5705	
Numbers of NIP‐funded vaccinations in the practice	2654	2864	
NIP‐funded vaccine coverage in practice in % (95% CI)	55.0% (53.6%–56.4%)	50.2% (48.9%–51.5%)	
Incidence rate ratio (intervention/control) of vaccination coverage (95% CI) in practice	1.096 (1.039–1.156)		< 0.001
Relative change (%) of vaccination coverage in % (95% CI) in the intervention practices in comparison to control	9.6% (3.9%–15.6%)		
B. Eligible group: Children 6 months to < 5 years			
Number of visits to the practice	1393	1042	
Numbers of NIP‐funded vaccinations in the practice	276	89	
NIP‐funded vaccine coverage in practice in % (95% CI)	19.8% (17.7%–22.0%%)	8.5% (6.9%–10.4%)	
incidence rate ratio (intervention/control) of vaccination coverage (95% CI) in practice	2.320 (1.827–2.946)		< 0.001
Relative change (%) of vaccination coverage in % (95% CI) in the intervention practices in comparison to control	132.0% (82.7%–194.6%)		
C. Eligible group: Aboriginal and Torres Strait Islander patients > 6 months			
Number of visits to the practice	2447	358	
Numbers of NIP‐funded vaccinations in the practice	865	52	
NIP‐funded vaccine coverage in practice in % (95% CI)	35.3% (33.4%–37.3%)	14.5% (11.0%–18.6%)	
Incidence rate ratio (intervention/control) of vaccination coverage (95% CI) in practice	2.434 (1.840–3.220)		< 0.001
Relative change (%) of vaccination coverage in % (95% CI) in the intervention practices in comparison to control	143.4% (84.0%–222.0%)		
D. Eligible group: Pregnant women			
Number of visits to the practice	260	359	
Numbers of NIP‐funded vaccinations in the practice	61	31	
NIP‐funded vaccine coverage in practice in % (95% CI)	23.4% (18.4%–29.1%)	8.6% (5.9%–12.0%)	
Incidence rate ratio (intervention/control) of vaccination coverage (95% CI) in practice	2.716 (1.763–4.186)		< 0.001
Relative change (%) of vaccination coverage in % (95% CI) in the intervention practices in comparison to control	171.6% (76.3%–318.6%)		

Abbreviations: NIP, National Immunisation Program; 95% CI, 95% confidence interval.

Among pregnant women, the NIP‐funded vaccination coverage was significantly higher in the intervention practices than in the control practices (23.5% vs. 8.6%; respectively; *p* < 0.001) (Table 5).

While estimating differences in vaccination coverage in different subgroups in the intervention practices compared to the control practices, we found significantly higher vaccination coverage for all four eligible subgroups. In pregnant women, NIP‐funded vaccination within practices is 2.7 times (95% CI: 1.8 to 4.2 times) higher in the intervention practices than in the control practices. In the Aboriginal and Torres Strait Islander cohort aged more than 6 months subgroup, the vaccination coverage is 2.4 times (95% CI: 1.8 to 3.2 times) higher in the intervention practices than in the control practices. In 6 months to less than 5 years age group, the vaccination coverage is 2.3 times (95% CI: 1.8 to 2.9 times) higher in the intervention practices than in the control practices. However, the differences in vaccination coverage are smaller but statistically significant (*p* value = 0.006) in the 65 years and older age group estimating only 1.1 times higher (95% CI: 1.0 to 1.2 times) in the intervention practices than in the control practices (Table 5).

## 4 Discussion

To the best of our knowledge, this is the first study to investigate the use of CDSS technology with influenza vaccine providers in medical practices in Australia. The ‘PenCS Flu Topbar’ app was successfully used by practices that were already actively using PenCS Topbars [16] prior to the introduction of the PenCS Flu Topbar app. They reported that it was easy to use, and it helped to identify patients who had not previously been identified as eligible for NIP‐funded flu vaccine. Practices that were not actively using PenCS Topbar apps prior to the introduction of the ‘PenCS Flu Topbar’ app did not report using it and did not report changing their usage pattern of Topbar following the app rollout. This study demonstrated that the use of CDSS was associated with increased influenza vaccine uptake.

The pilot impact evaluation found a significantly higher NIP‐funded seasonal influenza vaccination coverage in the practices where the ‘PenCS Flu Topbar’ app was deployed. The higher coverage was observed for all age groups and pregnant women. Conversely, the non–NIP‐funded vaccination coverage was lower in the practices where the app was used compared to those practices where the app was not used. It is unclear why non–NIP‐funded vaccination coverage was lower in the practices where the PenCS Flu Topbar app was used compared to those practices where the app was not used. One possible explanation is that GPs and PNs (of control areas) are recommending vaccination to a wider range of people, rather than relying solely on CDSS technology like PenCS Topbars. However, the improvement of NIP‐funded vaccinations after using CDSS tools like PenCS is consistent with some previous studies conducted in the United States and Australia [9, 17]. For instance, the Project IMPACT Immunisations Pilot conducted in Western Australia demonstrated that when pharmacists had access to immunisation information systems data and clinical decision‐support resources, they were able to identify additional vaccination opportunities and increase the number of administered routine vaccines by 41.4% [9, 17]. Incorporating CDSS technology like PenCS Topbar app is becoming increasingly important in healthcare, and hospitals are finding it is time to step up their CDS strategy, especially since clinical decision support is part of the meaningful use matrix.

Missed opportunities for vaccination occur when a patient presents at a medical practice but does not receive the vaccines they are due, and CDSS technology can improve adherence to clinical guidelines and vaccination rates [18]. While health technology is utilised with success using patient‐focused recall and reminders for on‐time vaccination, especially in infants, such as SMS reminders [19, 20, 21], it is a provider‐centred initiative focusing on recommending vaccines, consistently at all possible clinical encounters to improve vaccination uptake. Most of the medical practices in our study relied on their own method of recalling patient's vaccination history; however, there is evidence that CDSS technology can improve vaccination rates [22]. These missed opportunities for influenza vaccination occur when a provider may not be thinking about vaccination, be aware that a patient was unvaccinated, or eligible for vaccination [8]. Strategies to increase vaccination during routine GP visits such as CDSS technology can be used to target vulnerable populations and improve vaccination rates in target groups. Studies have demonstrated the effectiveness of such rule‐based algorithms in delivering accurate vaccine recommendation for childhood immunisation [23] and paediatric emergency settings [24].

CDSS technology not only positively impacts vaccine uptake but also reduces clinical burden. CDSSs can improve immunisation rates when integrated into routine workflow, implemented wherever care is delivered and used by all providers who administer vaccines [25]. CDSS automates eligibility screening, reduces inefficiencies and improves patient safety and outcomes for nurses and GPs [9]. The CDSS tool should ensure a significantly higher uptake of indicated immunisations, for the right patient populations, at the right time [23]. However, the effectiveness of the CDSS tool depends on many factors including usability, training and culture. Quality audits at regular intervals, user feedback and education can further improve the current mechanism, along with sending text reminders to eligible individuals/families [9, 26]. Further large‐scale studies are essential to determine the effectiveness of CDSS technology in improving vaccination uptake and the factors that enhance the effectiveness of harnessing CDSS.

Several factors have been reported to be associated with the uptake of CDSS applications. Jones et al. [27] stated that the uptake of CDSS depends on trustworthiness of the application. The authors [27] argued that the developers should ensure that the mechanisms and the sources of information that the system follows to come to a conclusion are transparent to the users. Earlier studies [28, 29] demonstrated that there is a strong relationship between the perceived usefulness of CDSS and the intention to use, underscoring the importance of generating patient‐specific and service‐specific outcomes of CDSS. Jansen‐Kosterink et al. [28] reported that perceived service benefits improve CDSS uptake while perceived risks of CDSS (e.g., threat to professional autonomy) reduce its uptake. The authors [28] found that interference with clinical practice, costs, time and system malfunctioning are the key reasons for not adopting CDSS. Also, the involvement of end‐users early in the design and throughout the development process is crucial to improving acceptance and uptake of CDSS [30].

Our evaluation has some limitations. The sample size for the quantitative study component was not adequate to generalise the findings at a national level. Selection of intervention and control practices was not random; hence, we could not minimise the chance of selection bias. Data were available only for a limited time frame (e.g. 1‐year postimplementation and 3‐year pre‐implementation data), and the number of medical practices was limited. This limited the ability to perform precomparison to postcomparison analysis. We could not perform further statistical analysis (i.e., interrupted time series or difference in differences analysis) with the available data. As the data were aggregated, comments cannot be made on confounding factors (e.g., age and sex) that could potentially influence the result. The vaccination coverage is estimated among the number of visits rather than the number of individuals visiting the practices, yet an individual could visit the practices multiple times in that time frame. However, the coverage estimation process was consistent for both intervention and control practices. Data are not available in this study to investigate why vaccination coverage for non‐eligible people was lower in the intervention practices than in the control practices.

While a reasonable level of thematic saturation was reached among the 11 practices that participated in the qualitative phase of the study, and the data can therefore be used to provide a good approximation of the broad views and experiences of key users of the PenCS Topbar apps in the CQ region, there may be views and experiences unique to those working in practices in other areas that were not captured in this sample.

Of important note, the timing of this evaluation overlapped with the COVID‐19 vaccine rollout in medical practices throughout Australia. This evaluation did not consider the effect COVID‐19 vaccine rollout on influenza vaccination uptake and app usage.

## 5 Conclusion

Our study evaluating the pilot implementation of ‘PenCS Flu Topbar’ app in CQ medical practices indicates that the introduction of the app is positively accepted by the users in medical practices. The app helps GPs, PMs and PNs to easily identify patients eligible for NIP‐funded flu vaccination during their visits to the practices. Overall, effective use of ‘PenCS Flu Topbar’ app potentially improves the NIP‐funded influenza vaccination coverage in other Australian regional areas similar to CQ. However, further evaluation is required to estimate the impact of implementation on a larger scale. More frequent and active use of ‘PenCS Flu Topbar’ app in the practices will produce more reliable results.

## Author Contributions

**Gulam Khandaker:** Conceptualization; Funding acquisition; Investigation; Methodology; Project administration; Writing – review and editing. **Gwenda Chapman:** Data curation; Investigation; Project administration; Writing – review and editing. **Arifuzzaman Khan:** Investigation; Methodology; Project administration; Software; Visualization; Writing – original draft; Writing – review and editing. **Mahmudul Hassan Al Imam:** Data curation; Resources; Software; Formal analysis; Writing – review and editing. **Robert Menzies:** Supervision; Validation; Visualization; Writing – review and editing. **Nicolas Smoll:** Formal analysis; Investigation; Methodology; Software; Writing – review and editing. **Jacina Walker:** Investigation; Resources; Supervision; Visualization; Writing – review and editing. **Michael Kirk:** Project administration; Resources; Supervision; Writing – review and editing. **Kerrie Wiley:** Data curation; Formal analysis; Methodology; Software; Visualization; Writing – review and editing.

## Ethics Statement

Ethical approval was obtained from the Townsville Hospital and Health Service Human Research Ethics Committee (HREC) (HREC/QTHS/73995 and HREC/QTHS/74743).

## Consent

Written informed consent was obtained from all participants.

## Conflicts of Interest

The authors declare no conflicts of interest.

### Peer Review

The peer review history for this article is available at https://www.webofscience.com/api/gateway/wos/peer‐review/10.1111/irv.13280.

## Supporting information


**Data S1.** Semi‐structured interview schedule.
**Data S2.** Data collection sheets for quantitative project component; overall numbers.
**Data S3.** Data collection sheets for quantitative project component; numbers in different subgroups.

## Data Availability

Research data can be accessed by contacting the corresponding author.
