# Supplementary material for: Evaluating Pilot Implementation of ‘PenCS Flu Topbar’ App in Medical Practices to Improve National Immunisation Program–Funded Seasonal Influenza Vaccination in Central Queensland, Australia
Source: Influenza Other Respir Viruses. 2024 Apr 16;18(4):e13280. doi: 10.1111/irv.13280 (PMC11019295; doi:10.1111/irv.13280)
Supplement: Supplementary file 1 — Data S1. Semi‐structured interview schedule. Data S2. Data collection sheets for quantitative project component; overall numbers. Data S3. Data collection sheets for quantitative project component; numbers in different subgroups. [file IRV-18-e13280-s001.docx]

**S1 Semi-structured interview schedule**

**Interview schedule – ‘PenCS Flu Topbar’ App pilot project INITIAL BASELINE INTERVIEWS**

**Orientation**

Tell me a little about your role here.

Probe for involvement with PenCS use.

**Influenza vaccination-specific activities**

Can you walk me through the process flu vaccination in your practice?

Probe for:

- how at-risk patients are currently identified for flu vaccine
- whether patients are actively recalled, e.g. by SMS, and the process for this
- how Pen CS system currently fits into the described process, probe for detail on how system is currently used in this context including any use of free-text notes

What do you think works well with this process? Probe for PenCS-specific feedback.

What do you think could improve? Do you have any ideas about how to do that? Probe for PenCS-specific issues.

Is there anything else you think is useful to know about how flu vaccines are given in your practice?

**Interview schedule – ‘PenCS Flu Topbar’ App pilot project POST-INTERVENTION INTERVIEWS**

Can you walk me through the process for flu vaccination in your practice with the ‘PenCS Flu Topbar’ app? How is it different to what it was before (if mapped previously we can use the previous process flow diagram as a discussion anchor point)

Tell me what you think about the ‘PenCS Flu Topbar’ app.

Probe for:

- reasons for their thoughts
- was it easy to use? How?
- was there anything that was difficult or annoying about it? Can you tell me about it?
- what did you think about the prompts? Did it change how you do things? What did it change? How did it change for you?
- Do you think it changed patient outcomes in your practice? How?
- Was there any feedback from your patients on the information they received? What was it? Did you notice any changes in the discussions you were having about flu vaccines with those patients (probe: do you think it helped?)
- Is there anything else you’d like to tell me about the app?

**S2 Data Collection sheets for quantitative project component; overall numbers**

| **Question** | | **Comments** | **Count** |
| --- | --- | --- | --- |
| Q1 | **How many people coming for consultation were eligible for the free vaccine?** | Enter the total of the 'Strongly Recommended and Covered by NIP' column in the Patient Groups (NIP) report regardless of the immunisation status |  |
| Q2 | **How many of the eligible individuals received flu vaccine at the practice?** | Enter the count for 'Influenza vaccine Received at the clinic' of the 'Strongly Recommended and Covered by NIP' column in the Patient Groups (NIP) report |  |
| Q3 | **How many of the eligible individuals received** flu **vaccine elsewhere?** | Enter the count for 'Influenza vaccine Received Elsewhere' of the 'Strongly Recommended and Covered by NIP' column in the Patient Groups (NIP) report |  |
| Q4 | **How many people visited the practice for consultation in the selected date range (1/03/2021 - 31/07/2021)?** | Enter the 'Population' from the title of the Patient Groups (NIP) report |  |
| Q5a | **How many of the NON-eligible individuals received flu vaccine at the practice?** | Enter the count for 'Influenza vaccine Received at the clinic' of the 'Strongly Recommended and But Not Covered by NIP' column in the Patient Groups (NIP) report |  |
| Q5b |  | Enter the count for 'Influenza vaccine Received at the clinic' of the 'Recommended and But Not Covered by NIP' column in the Patient Groups (NIP) report |  |
| Q6a | **How many of the NON-eligible individuals received flu vaccine elsewhere?** | Enter the count for 'Influenza vaccine Received Elsewhere' of the 'Strongly Recommended and But Not Covered by NIP' column in the Patient Groups (NIP) report |  |
| Q6b |  | Enter the count for 'Influenza vaccine Received Elsewhere' of the 'Recommended and But Not Covered by NIP' column in the Patient Groups (NIP) report |  |

**S3 Data Collection sheets for quantitative project component; numbers in different subgroups**

| **Question** | | **Comment** | **Children**  **6 months to 5 years** | **Adults**  **65 years and older** | **Pregnant patients** | **Indigenous patients**  **Over 6 months** |
| --- | --- | --- | --- | --- | --- | --- |
| Q7 | **How many patients received an influenza vaccine in 2021?** | Load the extract from (or just after) August 2021. After applying the respective filter, enter the total number for **'Current Year'** from the Influenza/General report in each column |  |  |  |  |
| Q8 | **How many patients who received the vaccine at the clinic were eligible for the free vaccine based on their age/ethnicity or pregnancy?** | Load the extract from (or just after) August 2021. After applying the respective filter, enter the count for **'Influenza vaccine Received at the clinic'** of the 'Strongly Recommended and Covered by NIP' column in the Patient Groups (NIP) report |  |  |  |  |
| Q8a | **How many patients visited the practices were eligible for the free vaccine based on their age/ethnicity or pregnancy in 2021?** | Load the extract from (or just after) August 2021. After applying the respective filter, enter the **total** of the 'Strongly Recommended and Covered by NIP' column in the Patient Groups (NIP) report regardless of the immunisation status |  |  |  |  |
| Q9 | **How many patients who received the vaccine elsewhere were eligible for the free vaccine based on their age/ethnicity or pregnancy?** | Load the extract from (or just after) August 2021. After applying the respective filter, enter the count for **'Influenza vaccine Received Elsewhere'** of the 'Strongly Recommended and Covered by NIP' column in the Patient Groups (NIP) report |  |  |  |  |
| Q10 | **How many patients received an influenza vaccine were eligible for the free vaccine based on their age/ethnicity or pregnancy in 2020?** | Load the extract from (or just after) August 2020. After applying the respective filter, enter the **total** of **'Influenza vaccine Received at the clinic'** and **'Influenza vaccine Received Elsewhere'** of the 'Strongly Recommended and Covered by NIP' column in the Patient Groups (NIP) report |  |  |  |  |
| Q10a | **How many patients visited the practices were eligible for the free vaccine based on their age/ethnicity or pregnancy in 2020?** | Load the extract from (or just after) August 2020. After applying the respective filter, enter the **total** of the 'Strongly Recommended and Covered by NIP' column in the Patient Groups (NIP) report regardless of the immunisation status |  |  |  |  |
| Q11 | **How many patients received an influenza vaccine were eligible for the free vaccine based on their age/ethnicity or pregnancy in 2019?** | Load the extract from (or just after) August 2019. After applying the respective filter, enter the **total** of **'Influenza vaccine Received at the clinic'** and **'Influenza vaccine Received Elsewhere’** of the 'Strongly Recommended and Covered by NIP' column in the Patient Groups (NIP) report |  |  |  |  |
| Q11a | **How many patients visited the practices were eligible for the free vaccine based on their age/ethnicity or pregnancy in 2019?** | Load the extract from (or just after) August 2019. After applying the respective filter, enter the **total** of the 'Strongly Recommended and Covered by NIP' column in the Patient Groups (NIP) report regardless of the immunisation status |  |  |  |  |
| Q12 | **How many patients received an influenza vaccine were eligible for the free vaccine based on their age/ethnicity or pregnancy in 2018?** | Load the extract from (or just after) August 2018. After applying the respective filter, enter the **total** of **'Influenza vaccine Received at the clinic'** and **'Influenza vaccine Received elsewhere’** of the 'Strongly Recommended and Covered by NIP' column in the Patient Groups (NIP) report |  |  |  |  |
| Q12a | **How many patients visited the practices were eligible for the free vaccine based on their age/ethnicity or pregnancy in 2018?** | Load the extract from (or just after) August 2018. After applying the respective filter, enter the **tota**l of the 'Strongly Recommended and Covered by NIP' column in the Patient Groups (NIP) report regardless of the immunisation status |  |  |  |  |
